# Evaluation of Risk Factors for the Occurrence of Limb Fractures in Children Due to Unintentional Injury in Podgorica, Montenegro, Mediterranean

**DOI:** 10.3390/medicina60010129

**Published:** 2024-01-10

**Authors:** Katarina M. Raspopovic, Dragic Bankovic, Aleksandra Petrovic, Valentina Opancina, Simon Nikolic, Radisa H. Vojinovic

**Affiliations:** 1 Institute for Children’s Diseases, Clinical Center of Montenegro, 81000 Podgorica, Montenegro; k.raspopovic@gmail.com; 2 Faculty of Sciences, University of Kragujevac, 34000 Kragujevac, Serbia; dragic@kg.ac.rs; 3 Clinic for Orthopedic Surgery and Traumatology, University Clinical Center of Serbia, 11000 Belgrade, Serbia; aleksandra.petrovic@kcs.ac.rs; 4 Department of Radiology, Faculty of Medical Sciences, University of Kragujevac, 34000 Kragujevac, Serbia; rhvojinovic@medf.kg.ac.rs; 5 University Clinical Center Kragujevac, 34000 Kragujevac, Serbia; 6 Faculty of Medicine, University of Kosovska Mitrovica, 11000 Belgrade, Serbia; simon.nikolic@med.pr.ac.rs

**Keywords:** fractures, unintentional injuries, children, risk factors, Balkans

## Abstract

*Background and Objectives*: Limb injuries in childhood are very common, with most of them being unintentional and often accompanied by soft tissue injuries. The aim of our study was to determine the risk factors that contribute to the occurrence of limb fractures as the most common type of accidental injury to children in our conditions. *Materials and Methods*: This study was designed as a prospective clinical analysis of predictive factors with a “nested” case–control study. It included all patients under the age of 18 who were diagnosed with unintentional limb injury and limb fracture due to accidental injury, at the Clinical Center of Montenegro, Podgorica, in the period of 7 January 2020–30 June 2021. *Results*: The gender of the child and the occurrence of the fracture are not related, and a statistically significant relationship was found between the occurrence of the fracture and the place of residence, the child’s age, body mass index (BMI), the affected limb, the method of injury, and the mental state of the parents of the injured child, as well as their economic status. It was proved that the older the child was, the lower the chance of injury, while multivariate analysis proved that BMI could be a predictor of accidental fracture. The most common method of accidental limb fractures in children was a fall from a height. *Conclusions*: The analysis of factors that influence the occurrence of children’s injuries is of great importance for public health. Such and similar research can enable a better understanding of the factors that influence accidental injuries, and therefore influence the prevention of these injuries by organizing various educational materials at the primary healthcare level or at the school level, for both children and parents.

## 1. Introduction

Injuries in children can occur accidentally, without prior intention, or intentionally (murder, interpersonal violence, wars and other forms of collective violence, suicide and other forms of self-harm) [1]. Unintentional injuries can be accompanied with soft tissue and/or organ injuries, as well as with or without bone fractures. The most common are fractures of the long bones of the upper (more often) and lower extremities [2]. Injuries occur very often in childhood, and fractures account for 10–25% of all injuries [3]. According to the data, the total incidence of fractures in children is 20.2/1000/year [4], which is twice as much compared to the adult population [5]. It can be said that the incidence of fractures increases with age, and that about one third of children will suffer a fracture by the age of 16 [2]. About 830,000 children die from accidental injuries every year, and about 95% of those deaths occur in low- and middle-income countries [6]. This presents a significant economic and public health burden for these countries, but it is also an important problem at the global level. In high-income countries, risk factors and protective factors for the occurrence of injuries in children have been identified [7]. Numerous factors influence the occurrence of injuries and/or fractures and can be divided in different ways. The most frequently mentioned are gender, age, socio-economic differences, obesity, and low bone mineral density (BMD) and are factors related to the child itself [8,9,10]. Environmental factors include time of day, seasonal and climatic differences, home environment, school environment, cultural, and other differences [11,12]. It is estimated that most of the accidental injuries in childhood occur in the home and immediate environment, where children are believed to be safe [13]. A large number of children’s injuries (44%) occur at school and near school [14], such as traffic trauma, as passengers or pedestrians [15,16,17,18]. Playing sports can often be the cause of fractures in children, with an incidence of 5.63/1000/year; boys are injured much more often than girls (87%:13%), and 84% of fractures are related to the upper extremities [17].

Until now, there are no published papers on the topic of risk factors that contribute to the occurrence of limb fractures in children, in our region. Due to the high occurrence of this type of pathology, there is great clinical interest in this field and a great need for epidemiological studies with this topic in the future.

The aim of our work was to determine the risk factors that contribute to the occurrence of limb fractures as the most common type of accidental injury to children in our conditions, considering the frequency of accidental injuries and fractures, as well as their impact on the child, family members, and the community.

## 2. Materials and Methods

### 2.1. Study Design and Study Population

This study was designed as a prospective clinical study of predictive factor analysis with a nested case–control study. The study documentation was approved by the Ethics Committee of the Clinical Center of Montenegro, decision number 03/01-13995/1, dated 19 July 2018.

It included all patients < 18 years of age who were diagnosed with accidental limb injury and limb fracture due to accidental injury, at the Clinical Center of Montenegro, Podgorica, in the period of 7 January 2020–30 June 2021. Clinical examinations of children were performed by pediatric orthopedists, pediatric surgeons, or orthopedists, who also determined the indications for radiological imaging of patients. The Clinical Center of Montenegro in Podgorica is a tertiary-level institution, the only one of its kind in Montenegro. Podgorica is the capital with about 190,000 inhabitants, and the whole of Montenegro has about 620,000 inhabitants.

Necessary data for conducting this study were obtained from the medical documentation of the patients and from the survey of patients or parents (shown in Supplement Table S1, created by authors), also including the treatment that was carried out and the diagnostic methods that were used. Data that were included, apart from clinical data, were related to the diagnosis of the injury itself and/or fracture, gender, age, height and weight, without clothes and shoes (body mass index—BMI), place of residence (village or city), method of injuries (fall on a flat surface; fall from a height; traffic accident in a motor vehicle; injury to a pedestrian; injury on another means of transport such as a bicycle, scooter, skateboard; other). The following information was collected from the parents via a questionnaire: mother’s age, father’s age, whether the father or mother is being treated or has been treated for depression and/or anxiety, parents’ employment (unemployed; one employed; two employed), and the family’s economic status according to personal understanding (low income; middle income; high income). These were the criteria that the patient had to fulfill in order to be included in this study. The exclusion criteria were (1) injuries and fractures of extremities caused by intentional injury by the patient themself; (2) injuries and fractures of the extremities caused as a result of intentional injury by another person (physical violence, sexual assault, stab wounds, gunshot wounds); (3) injuries and fractures of extremities caused by the use of alcohol or other addictive substances; (4) non-cooperation of children for any reason; (5) severe head injuries; and (6) patients with incomplete relevant data in medical and other documentation.

Patients were divided into two groups. The first group consisted of patients in whom the imaging-proven fracture of the extremity was caused by an unintentional injury (group of cases). The second group consisted of patients with an unintentional injury of the extremity, in whom a fracture was ruled out by radiological diagnosis as part of the clinical assessment (control group).

### 2.2. Statistical Analysis

Statistical analysis was performed using SPSS Version 19 (IBM SPSS Statistics, New York, NY, USA). The Kolmogorov–Smirnov test and Shapiro–Wilk test were used to test the normal distribution. Comparisons between two groups were analyzed by the Mann–Whitney U test. Qualitative data were compared between two groups using the chi-square (χ^2^) test. Continuous variables are presented as median (25th percentile–75th percentile) and qualitative variables as *n* (%). Univariate and multivariate binary logistic regressions, including odds ratio (OR), were performed to determine the effects of each factor on the dependent variable (fracture). A receiver operating characteristic (ROC) curve was generated and the area under the curve was calculated. This method was used to investigate the prognostic value of variable BMI. The sensitivity and specificity for optimal cut-off were calculated. Differences were considered significant at *p* < 0.05.

## 3. Results

A total of 320 patients were included in this study, out of which 80 were children with an accidental injury and fracture of an extremity, and 240 were children with an accidental injury without a fracture. The baseline characteristics of the participants in this study are given in Table 1.

**Figure 1:**
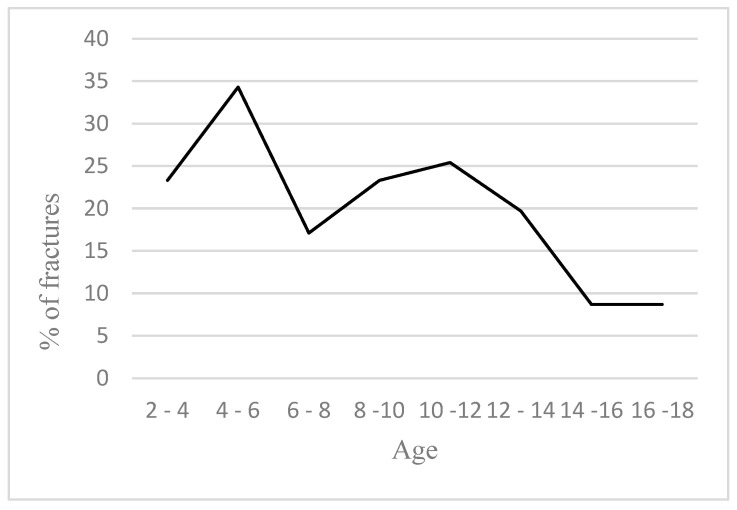
Percentage of fractures according to the age of children.

The gender of the child and the occurrence of fracture were not related (*p* = 0.458). Children from the countryside had a higher percentage of fractures (48.2%) than children from the city (20.2%). The difference in age between children with injuries and children with fractures was statistically significant (*p* = 0.006). The average age of children with fractures was 9.92 (5.87–12.37) years, and that of children with injuries without fractures was 11.50 (7.25–14.12) years. Fractures occurred more often in children who were younger than 11 (32.2%), compared to children who were 11 years or older (19.0%). The percentage of fractures (in relation to injuries) according to the age of children is presented in Figure 1. The difference in BMI between children with injuries and children with fractures was statistically significant (*p* < 0.001). Children with injuries had a BMI of 17.95 (16.35–19.95) and children with fractures had a BMI of 21.10 (19.70–24.80).

Children are usually considered to be normally nourished if their BMI is less than the 85th percentile, overnourished if it is between the 85th and 95th percentile, and obese if it is greater than or equal to the 95th percentile. In our sample, the 85th percentile BMI was 21.2 and the 95th percentile was 25.3. The occurrence of fractures was more common in overfed children (71.4%) than in children who were normally fed (15.3%), and was most frequent in obese children (100.0%). The percentage of fractures in the upper extremities was 34.4%, while that in the lower extremities was 10.4%, which was proven to be statistically significant. The occurrence of fractures was related to the method of injury. When falling from a flat position, the percentage of fractures was 22.4%, that when falling from a height was 48.2%, while that for other methods was 11.9%. If a parent had depression and/or anxiety, the percentage of fractures in children was significantly higher than in children whose parents did not have any of these diagnoses (69.2% vs. 23.1%). In children with one parent that was employed, the percentage of fractures was 34.5%, and in children with both parents that were employed, the percentage of fractures was 19.8%. The occurrence of fractures in children was related to the economic status of the family. In children from families with low incomes, the percentage of fractures was 33.5%, that with middle incomes was 17.6%, and that with high incomes was 12.9% (Table 2).

Univariate binary logistic regression showed that the occurrence of fracture was influenced by children’s age, rural life, BMI, low household income, employment of both parents, and parental depression and/or anxiety. The older the child, the lower the chance of fracture. Living in the countryside, low family income, and parental depression and/or anxiety increased the likelihood of fracture, while the employment of both parents decreased the likelihood of fracture. Multivariate binary logistic regression showed that the onset of fracture was influenced by children’s age, BMI, and parental depression and/or anxiety. A child’s older age reduced the possibility of a fracture, while a higher BMI increased the possibility of a fracture. Children whose parents had a diagnosis of depression and/or anxiety had a 25 times greater chance of fracture than children whose parents did not have any of these diagnoses (Table 3).

**Figure 2:**
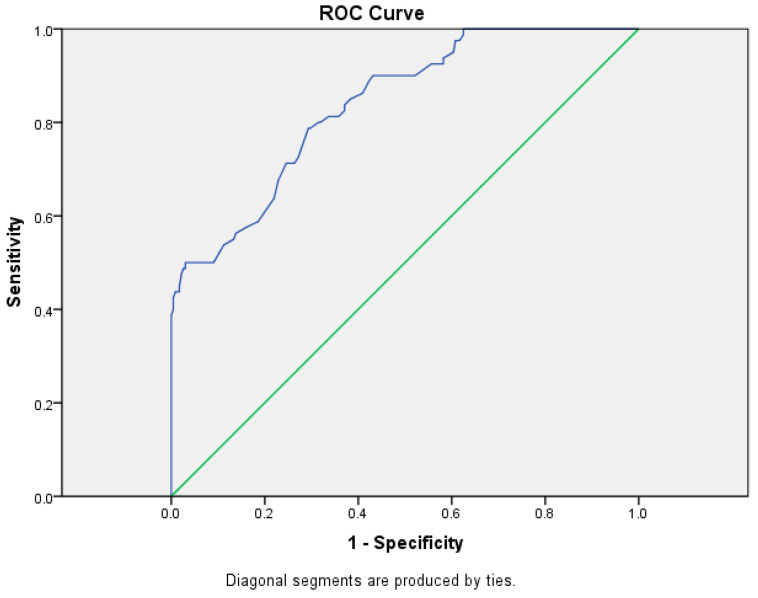
ROC curve for BMI.

BMI was proven to be a significant predictor of fracture occurrence. In the following part, it was examined whether BMI can be a “marker” for the occurrence of fractures. The area under the ROC curve (AUROC) showed that BMI can be an excellent “marker” for fracture occurrence (AUROC = 0.838, *p* < 0.001). The optimal cut-off was 19.6. We considered that BMI, as a “marker”, was negative if it was less than 19.6 and positive if it was greater than or equal to 19.6. The sensitivity was 0.79 and the specificity was 0.71, which means that 79% of children with a fracture had a BMI ≥19.6 and 71% of children with an injury had a BMI <19.6 (Figure 2).

## 4. Discussion

Numerous studies indicated a large number of risk factors for the occurrence of unintentional injuries in childhood. In our study, we tried to identify the main risk factors for the occurrence of fractures in the case of unintentional injury to children in Podgorica, the capital of Montenegro, a small country in the Mediterranean belt, which belongs to the group of low- and middle-income countries (LMICs). At the same time, we wanted to determine the methods of injury for the unintentional limb fractures in children. Among the injured children, there were almost twice as many boys, which is in accordance with the literature data [14,19,20,21]. There was a higher percentage of fractures among boys than girls, but this was not statistically significant. It could be concluded that in our study, boys were injured more often than girls, but that the outcome of injuries in terms of fractures was similar in both genders. Birgul et al. in their study state that the proportion of boys compared to girls among all injured was 75.3%:24.7%. If the ratio of male to female children with fractures was analyzed, this difference disappeared (boys with fractures/girls with fractures = 28.9%:31%) [14]. This is in agreement with the results of our study. However, most of the other studies report that the male gender predominates in children with a fracture. Aygun et al. analyzed 1020 children and reported that boys had fractures three times more often in comparison to girls, and this study analyzed fractures of all regions [22]. Similar results were reported by other authors [3,4,23,24,25,26].

Analysis of the home location showed that children living in the countryside had a much higher percentage of accidental fractures than children living in the city (48.2%:20.2%). Univariate binary logistic regression showed that living in the countryside increases the possibility of unintentional fractures in children (*p* < 0.001). This can be partly explained by the poorer living conditions [27,28], but it should also be taken into account that half of the injured children from the countryside had a fracture (the ratio of children from the city is injury/fracture = 4:1). One of the conclusions can be that children who live in the countryside are taken to the doctor only when a serious injury is involved. Fractures occurred more often in children younger than 11 years, compared to children aged 11–18 years (32.2%:19.0%), and children with fractures are younger than children with injuries by about 1.7 years. According to the obtained results of univariate and multivariate binary logistic regression, the older the child, the lower the chance of accidental fractures (*p* = 0.003; *p* < 0.001).

A large study by Kessler et al. (2013) indicated that fractures in children most often occur between the ages of 6 and 11 [29]. Similar results were reported by many other authors [22,30,31,32]. Halawa et al. in their study found that the highest percentage of injuries was in the age group of 2 to 6 years, being 67.5% [23]. When analyzing specific methods of injury (sports, bicycle, pedestrians, playgrounds), studies report on different age groups that are injured more often, with the occurrence of fractures. Sports are more often played by children of school age, so this research was focused on these age categories [12,17,24]. Beckwith et al. reported that 39.7% of children with a fracture (which occurred due to bike riding) were aged 10–16 years, similar to Pardi et al.’s results [33,34]. Joeris et al. reported that traffic-related fractures were most common in children aged 11–17 years old, which was similarly proven by Mulugeta et al. [24,35]. Playgrounds are mostly occupied by children of preschool age, so injuries and fractures that took place there are related to the children up to 6 years old [24,36]. The claim of some authors that at least one third of children experience a fracture by the age of 16 or before the age of 17 is very worrying [2,3]. Many studies showed that BMI is a significant factor in the occurrence of injuries and fractures in children. Our results showed that the BMI of children with fractures was significantly higher than the BMI of children with injuries without fractures, and that the percentage of fractures was significantly higher in overfed and obese children compared to normally fed children.

Univariate binary logistic regression confirmed that an increase in BMI influenced the occurrence of accidental fractures, and multivariate binary logistic regression has proven that overfed and obese children have a 12.5 times higher chance of accidental fracture than normally fed children. Univariate and multivariate binary logistic regression confirmed that an increase in BMI affected the increase in the frequency of accidental fractures. Similar results were obtained by numerous other studies [10,24,29,37,38,39,40]. Some studies did not show an association between the rise in BMI and the increased chance of fractures [25,41]. However, most authors agree that an increased BMI creates a greater chance of fractures in the extremities, which our study also showed. In our research, we additionally determined, using the ROC curve, that BMI was an excellent “marker” for the occurrence of accidental fractures in children (AUROC = 0.838, *p* < 0.001; cut-off = 19.6; the calculated sensitivity is 0.79, the specificity is 0.71, which is significantly high).

In our study, unintentional fractures of the upper extremities were significantly more frequent than those of the lower extremities (34.4%:10.4%). In the literature, the situation is very similar to our findings, so Aygun et al. reported that upper extremity fractures were three times more frequent than lower extremity fractures [22]. Similar results were reported by other authors [2,4,12,24,42]. A higher incidence of lower extremity fractures had been associated with specific activities in children’s playrooms [43] or with obesity [44]. Also, parental depression and/or anxiety, the employment of parents, and the economic status of the family showed statistical significance as risk factors. If parents had depression and/or anxiety, the percentage of fractures was three times higher. The application of univariate and multivariate binary logistic regression confirmed this as a significant factor, and it showed that the chance of fracture was about 25 times higher in children whose parents had depression and/or anxiety.

An analysis with similar results was provided by Baker et al. in a study in which they concluded that episodes of maternal depression and/or anxiety were associated with increased rates of child poisoning, fractures, and burns [45]. Other authors also proved the connection between parents’ mental illnesses and children’s injuries [46,47]. Our study showed that in cases where one parent is employed, there is a higher percentage of accidental fractures compared to families where both parents are employed (34.5%:19.8%). Univariate binary logistic regression showed that the chance of accidental fracture was reduced in cases with both parents being employed. At the same time, the economic status of the family affected the occurrence of accidental fractures in children in a similar way: fractures were most common in families with low income (33.5%), somewhat less common in families with a medium level of income (17.6%), and the rarest in families with high income (12.9%). This result was also confirmed by univariate binary logistic regression, which showed that low income increases the chances of accidental fractures in children (*p* = 0.001). These last two indicators (one parent that is employed and low income in the family) can also be logically reasoned, because it is common for a family with fewer employees to have a lower level of income.

Other studies report heterogeneous data on family environmental factors on the occurrence of injuries and fractures in children. A study from Egypt reported that children from low-income families were 38% more likely to be injured compared to middle- or high-income households [48]. This problem with the increased risk of injuries in families with lower incomes was also indicated in Iran [9]. Similar results were given in Germany by Foetinger et al. [49]. In a large number of studies, the mother’s employment was marked as a risk factor which increased the chances of children’s accidental injuries [9,23,27,50]. This was explained by the unemployed mother’s greater care for her children due to more free time. This was not the case in our study and we believe that additional research is needed to better define this problem, with the inclusion of numerous other parameters (influence of urbanity/rurality, economic status of the family, number of household members, childcare by grandparents, inclusion of children to preschool institutions—kindergartens, etc.).

The analysis of the methods of accidental injury and the occurrence of accidental fractures in children showed that falls can be classified into three different groups: falling from a flat surface, falling from a height, and other ways. In the first group (fall from a flat surface), the percentage of accidental fractures was 22.4%; in the group where the injury was caused by a fall from a height, 48.2% of accidental fractures occurred; and 11.9% of accidental fractures occurred in other ways. A study that covered a ten-year period and investigated accidental fractures of children in the USA from children’s playgrounds reported that in 49.8%, the cause was a fall from a height [51]. Rennie et al. in a study from Scotland concluded that a fall from a height of up to 1 m was the most common cause of accidental fractures in children, while Joeris et al. in Switzerland found that in 2716 children with accidental fractures, a fall was the most common cause in 27% [4,24]. Similar results were presented by many other studies that talk about accidental falls as the most common method of causing accidental injuries [14,19,20,35].

Unintentional injuries and limb fractures are common health problems of children all over the world, and the investigation of risk factors in these cases is important in order to reduce their occurrence. This would not only benefit the patients but also reduce the financial burden of healthcare. We encourage more, similar studies in this field in low- and middle-income countries.

## 5. Conclusions

Our study has shown that unintentional fractures occur more often in children younger than 11, as well as in those who live in the countryside. There was no difference in the distribution of fractures according to gender. Accidental fractures were more frequent in the upper extremities. The most important risk factor was an increased BMI, which has been proven to be an excellent “marker” for the occurrence of accidental fractures. Parental depression and/or anxiety, one parent that is unemployed, and a low level of family financial income also had an influence on the increase in accidental fractures. The most common cause of accidental limb fractures in children was a fall from a height.

## Figures and Tables

**Table 1:** Baseline characteristics of the study participants.

Characteristics	*n* (%) or Median (25th Percentile–75th Percentile)
Gender	
male	207 (64.7%)
female	113 (35.3%)
Residence	
city	264 (82.5%)
the countryside	56 (17.5%)
School	
not in school	56 (17.5%)
primary school	219 (68.4%)
High School	45 (14.1%)
Age	11.25 (7.25–13.83)
Height	144 (122.00–161.00)
Weight	40.50 (25.00–53.00)
BMI	18.90 (16.80–30.50)
Mother’s age	34.00 (29.00–38.00)
Father’s age	35.00 (31.00–39.00)

**Table 2:** Differences between injuries and fractures.

Variable	Category	Injury	Fracture	*p*
Gender	male	152 (73.4%)	55 (26.6%)	0.458
	female	88 (77.9%)	25 (22.1%)	
Residence	city	211 (79.9%)	53 (20.2%)	<0.001
	the countryside	29 (51.8%)	27 (48.2%)	
Age	less than 11 years	99 (67.8%)	47 (32.2%)	0.045
	11 years or older	141 (81.0%)	33 (19.0%)	
Obesity	normally fed	222 (84.7%)	40 (15.3%)	<0.001
	overfed	10 (28.6%)	25 (71.4%)	
	obese	0 (0.0%)	15 (100.0%)	
Extremity	upper	128 (65.6%)	67 (34.4%)	<0.001
	lower	112 (89.6%)	13 (10.4%)	
Mode of injury	fall from flat surface	159 (77.6%)	46 (22.4%)	<0.001
	fall from a height	29 (51.8%)	27 (48.2%)	
	other	52 (88.1%)	7 (11.9%)	
Parental depression and/or anxiety	without diagnosis	236 (76.9%)	71 (23.1%)	0.001
	with a diagnosis	4 (30.9%)	9 (69.2%)	
Employment of parents	unemployed	2 (100%)	0 (0%)	0.010
	one employed	76 (65.5%)	40 (34.5%)	
	two employed	162 (80.2%)	40 (19.8%)	
Economic status	low incomes	105 (66.5%)	53 (33.5%)	0.002
	middle incomes	108 (82.4%)	23 (17.6%)	
	high incomes	27 (87.1%)	4 (12.9%)	

**Table 3:** Influence of the examined factors on the occurrence of fracture.

	Univariate Binary Regression		Multivariate Binary Regression	
	Odds Ratio	*p*	Odds Ratio	*p*
Age	0.914 (0.862–0.970)	0.003	0.384 (0.283–0.522)	<0.001
The countryside	3.707 (2.025–6.784)	<0.001		
BMI	1.887 (1.599–2.228)	<0.001	12.521 (5.408–28.991)	<0.001
Low income	2.524 (1.487–4.283)	0.001		
Both parents are employed	0.469 (0.280–0.786)	0.004		
Parental depression and/or anxiety	7.479 (2.236–25.013)	0.001	24.871 (3.199–193.341)	0.002

## Data Availability

Data supporting the reported results are available on request from the corresponding authors.

## Supplementary Materials

The following supporting information can be downloaded at https://www.mdpi.com/article/10.3390/medicina60010129/s1, Table S1: Survey for patient and parents.
