# Supplementary material for: Evaluation of Risk Factors for the Occurrence of Limb Fractures in Children Due to Unintentional Injury in Podgorica, Montenegro, Mediterranean
Source: Medicina (Kaunas). 2024 Jan 10;60(1):129. doi: 10.3390/medicina60010129 (PMC10820898; doi:10.3390/medicina60010129)
Supplement: Supplementary file 1 [file medicina-60-00129-s001.zip › medicina-2755097-supplementary.pdf]

Supplement Table S1. Survey for patient and parents

Name and surname of the patient	
Name and surname of parents	
age of the patient	
date of birth of patient	
gender	Male/Female
residence	village/city
education	grades 1-4,<br>grades 5-8,<br>secondary school grades 1-4
height	
weight	
body mass index of the child (kg/m2)	
fracture location	
radiological report of the fracture	
way of injury	fall; traffic accident in a vehicle; pedestrian injury; injury on another vehicle such as a bicycle, scooter, skateboard;<br>other:___________________________________
location of the injury	home, school, training,<br>other:_______________________________
treatment	discharged after treatment, hospitalized—without or with surgery
outcome	complete recovery, no significant impairment of function, short-term impairment, long-term impairment, death,<br>other:_______________________________
complications	infection, sepsis, other: __________________________
absence from school after fracture (number of days):	
absence of physical activity after the fracture (number in days):	
inability to perform daily activities (specify which ones)	
concomitant diseases	diabetes, anemia, other:__________________
concomitant therapy	
sports activities	yes/no
type of sport and number of trainings per week	
previous accidental fracture	yes/no
psychiatric diagnosis	anxiety, depression, anorexia,<br>other:____________________
diet	vegetarians, vegans, religious fasting,<br>other:_______________________________
age of parents	mother________________ father______________
parents' education	uneducated, elementary school, high school, college education, doctorates
marital status of parents	married, divorced, widowed, other ___________
status of parents	two parents, single parent, foster family, adoptive parents, other:___________________

psychiatric diagnoses of parents	yes/no
accidental fractures of parents in childhood	yes/no
work status of parents	unemployed, one employed, both employed
income of parents	low, medium, high income
ISS score value	
PTS score value	
